# Hydrodesulfurization of Dibenzothiophene: A Machine Learning Approach

**DOI:** 10.1002/open.202400062

**Published:** 2024-04-12

**Authors:** Guadalupe Castro, Julián Cruz‐Borbolla, Marcelo Galván, Alfredo Guevara‐García, Joel Ireta, Myrna H. Matus, Amilcar Meneses‐Viveros, Luis Ignacio Perea‐Ramírez, Miriam Pescador‐Rojas

**Affiliations:** ^1^ Departamento de Química Universidad Autónoma Metropolitana-Iztapalapa Av. Ferrocarril San Rafael Atlixco 186, Col. Leyes de Reforma 1 A Sección, Iztapalapa C.P. 09310 Ciudad de México México; ^2^ Área Académica de Química Centro de Investigaciones Químicas – Universidad Autónoma del Estado de Hidalgo Carretera Pachuca-Tulancingo km. 4.5, Ciudad del Conocimiento C.P. 42184 Mineral de la Reforma, Hidalgo México; ^3^ Departamento de Química CONAHCYT-Universidad Autónoma Metropolitana-Iztapalapa Av. Ferrocarril San Rafael Atlixco 186, Col. Leyes de Reforma 1 A Sección, Iztapalapa C.P. 09310 Ciudad de México México; ^4^ Instituto de Química Aplicada Universidad Veracruzana Av. Luis Castelazo Ayala s/n, Col. Industrial-Ánimas, A.P. 575 Xalapa, Ver. México; ^5^ Departamento de Computación CINVESTAV-IPN Av. IPN 2508, Col. San Pedro Zacatenco, C.P. 07360 Ciudad de Mexico México; ^6^ Escuela Superior de Cómputo, Instituto Politécnico Nacional Instituto Politécnico Nacional Av. Juan de Dios Bátiz s/n, esq. Av. Miguel Othón de Mendizabal, Col. Lindavista, Gustavo A. Madero, C. P. 07738 Ciudad de México México

**Keywords:** Hydrodesulfurization, Computational chemistry, Lasso regression, Heterogeneous catalysis, Selectivity HYD/DDS

## Abstract

The hydrodesulfurization (HDS) process is widely used in the industry to eliminate sulfur compounds from fuels. However, removing dibenzothiophene (DBT) and its derivatives is a challenge. Here, the key aspects that affect the efficiency of catalysts in the HDS of DBT were investigated using machine learning (ML) algorithms. The conversion of DBT and selectivity was estimated by applying Lasso, Ridge, and Random Forest regression techniques. For the estimation of conversion of DBT, Random Forest and Lasso offer adequate predictions. At the same time, regularized regressions have similar outcomes, which are suitable for selectivity estimations. According to the regression coefficient, the structural parameters are essential predictors for selectivity, highlighting the pore size, and slab length. These properties can connect with aspects like the availability of active sites. The insights gained through ML techniques about the HDS catalysts agree with the interpretations of previous experimental reports.

## Graphical abstract

This work analyzes the hydrodesulfurization (HDS) process by compiling data on catalysts derived from MoS_2_, including their composition, structure, and experimental conditions. Using supervised machine learning methods such as regularized regression and random forest, the study estimates the conversion of DBT and the reaction selectivity. The results obtained allow the identification of an appropriate method for these estimations.

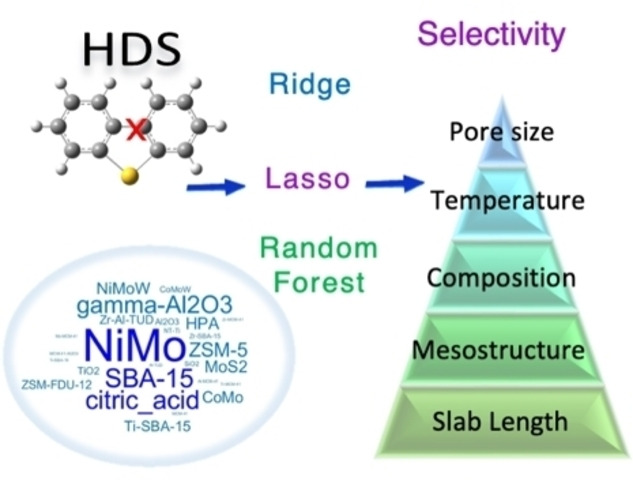

## Introduction

Obtaining clean energy with lower environmental impact has become crucial due to the increasing demand for energy and the need for practices that support the environment and the health of living beings.[^1^, ^2^, ^3^, ^4^] Despite the broad interest in using clean energy, fossil fuels are still the primary energy source. Consequently, the obtention of low sulfur fuel is of great interest. Moreover, most countries have regulated the sulfur content in the fuels. In some European and American countries, the sulfur content limits are 10 to 15 parts per million (ppm) for diesel, requiring efficient hydrodesulfurization processes.[^4^, ^5^, ^6^, ^7^, ^8^, ^9^] In several countries, the amount of light crude oil is decreasing; for this reason, heavy oil processing is necessary, which is challenging for current technologies.^[5]^ In this way, fuel desulfurization is a critical problem for fuel production and is relevant in the petrochemical industry. Therefore, diverse methods such as hydrodesulfurization (HDS), oxidative desulfurization, extractive desulfurization, adsorptive desulfurization, and biodesulfurization have been explored.^[8]^ However, the only method implemented in the industry for removing sulfur compounds is the HDS. This method employs a catalyst, high temperature (300–400 °C), and hydrogen gas pressure conditions.^[8]^

The principal HDS catalysts in use are bimetallic materials derived from the MoS_2_ and WS_2_, that include nickel and cobalt promoters. Initially, these materials were used in bulk, and supports like alumina were included in later years; most of these materials have low efficiency for removing refractory compounds like thiophene, dibenzothiophene (DBT), 4,6‐dimethyl dibenzothiophene (4,6‐DMDBT) and derivates, which are present significantly in heavy oil.^[4]^ In the early 2000s, the Nebula (New‐Bulk‐Activity) catalyst was developed, which is a trimetallic catalyst that showed a superior activity than the previous catalysts to perform deep HDS. Nevertheless, this catalyst is expensive and consumes a high amount of hydrogen, representing a clear disadvantage.[^7^, ^8^, ^9^, ^10^] Therefore, in the last decades, the interest in generating new catalysts to improve the efficiency of deep HDS for obtaining zero sulfur fuel has increased. One of the aspects that has attracted great attention is the modulation of the support‐catalyst interaction, which is connected to material properties like the dispersion of the catalysts in the support, surface area, pore size, slab length, and grade of stacking.^[11]^ It was suggested that the activity of HDS increases according to the surface area because the catalytic process occurs at the edges of the catalyst, therefore it is preferable to develop catalytic materials with a high number of active sites.^[4]^ Hence, to improve the catalytic ability, it has been modulated the number of active sites and their availability by including structure‐directing agents, which generate mesostructures and nanomaterials. Moreover, the use of additives and distinct impregnation methods could influence the reactivity of these materials and the interaction between catalysts and support.[^4^, ^12^] However, the structural characterization of these materials, which is fundamental for studying these processes, is challenging due to its complexity. Therefore, it is still necessary to understand the relation and impact of the mentioned characteristics of the catalytic materials on the HDS method.

The above catalyst properties are modulated to improve the performance of HDS. Several ways to evaluate this performance have been reported, such as the conversion of DBT, which represents the percentage of sulfur compounds removed. Another relevant aspect is the reaction mechanism of aromatic sulfur compounds. For instance, the HDS of DBT can be carried out by two different pathways: direct desulfurization (DDS) and hydrogenation (HYD). The first, DDS, breaks C−S bonds, leading to biphenyl (BP) generation. While HYD requires the pre‐hydrogenation of the aromatic ring followed by the elimination of the sulfur atom, the products are tetrahydrodibenzothiophene (THDBT), cyclohexenylbenzene (CHEB), and cyclohexylbenzene (CHB). The selectivity of HDS is determined by the distribution of products, specifically the relation between HYD and DDS products. This makes selectivity and conversion of DBT key indicators of catalyst efficiency and performance.^[12]^

Optimization, evaluation, and comprehension of many chemical processes are challenging due to the wide range of parameters and factors involved. Nowadays, machine learning (ML) algorithms could be a way of dealing with this multifactorial issue. These may help us to find key properties of the catalyst for deep HDS. For instance, Strieth‐Kalthoff et al. estimated the yield of organic reactions such as Buchwald–Hartwig, Suzuki couplings, and nucleophilic aromatic substitutions by ML.^[13]^ However, they found several limitations, such as the high dimensionality of databases needed due to the complexity of the chemical environment that involves reactants, condition reactions, synthesis techniques, and characterization of products, among others. Moreover, a loose homogeneity exists in the reported data, which can limit the generation of databases and ML implementation.^[13]^ Finally, it is worth mentioning that most publications only report successful experiments, implicating a lack of valuable information for training algorithms and process optimization.^[14]^ Therefore, creating databases with high quality for estimating the yield of chemical reactions is challenging. Consequently, the prediction of properties of chemical reactions is limited by the quality and quantity of pertinent descriptors. Some ML algorithms require considerable data (such as those based on neural networks) that could be unavailable for some chemical processes.[^15^, ^16^] In addition, the aspects above mentioned of a reaction limit some ML techniques, which leads to overfitting problems because of the high dimensionality and a limited number of registers. However, algorithms such as Random Forest and Regularized Regression can overcome these limitations and have been applied widely in related chemical studies.[^17^, ^18^, ^19^, ^20^, ^21^, ^22^]

In this contribution, the influence of descriptors such as composition, structure, reaction conditions, and catalyst synthetic aspects on the performance of catalysts for deep HDS was investigated by estimating the conversion of DBT and the catalyst selectivity (HYD/DDS). To reach this goal, the Random Forest method and regression regularized algorithms such as least absolute shrinkage and selection operator (Lasso) and Ridge were employed. A comparative analysis of each technique was done. A database was built by collecting information from scientific publications about catalysts for deep HDS. Moreover, the effect of the lack of data about low conversion on the algorithm performance and score is discussed, which is vital for obtaining correct estimations.

## Methods and Materials

To analyze the HDS of DBT, information about catalysts derived from MoS_2_ was gathered. These data served as a starting point to estimate the conversion of DBT and selectivity. The database contains data from scientific articles included in Scopus, Web of Science, and SciFinder. The details of the data selection are described in the supporting information (SI). In Table S1 of SI it is listed the sources consulted for each catalyst considered in this contribution. Published articles from 2005 until 2022 were considered because, after the development of Nebula catalyst (early 2000s), a new stage in the research dedicated to deep HDS started.^[10]^ The articles were chosen if they contained the properties shown in Table 1, which were defined to consider essential information describing the HDS process, such as composition, structural parameters of catalysts, and reaction condition. The composition predictors were determined by classes of atoms included in the catalyst, support, and kind corresponding mesostructure. The structural parameters considered were pore size, surface area, molybdenum atom dispersion, slab length, and grade stacking. It is expected that by taking into account these structural parameters one can indirectly describe the catalytic sites of the materials. Moreover, it was considered all the catalysts contained in each article. It is worth mentioning that the catalyst and support compositions are considered independently since most references analyze the effect of the support modification or the addition of promoters and additives to MoS_2_ separately. Furthermore, there are five principal catalysts, the monometallic (MoHet, where Het stands for heteroatom like S), two bimetallic (NiMo and CoMo), and two trimetallic (NiMoW and CoMoW). At the same time, the more representative supports are Al_2_O_3_, SiO_2_, TiO_2_, and their mesostructures.

**Table 1:** Predictor classification of HDS dataset.

Class data	Predictors in dataset	Number of predictors^[a]^
Reference	Author, Title, Source, DOI, Year publication, Abstract, Keywords	0
Composition	Kind_catalyst , Promoters, No_Metals , Metal_cat1 , Metal_cat2 , P_Mo , P_Ni , P_Co , P_W , Support_1 , Support_2 , Element_1 , Element_2 , Element_3 , P_Si , P_Al , P_Ti , Structure_directing_agent , Impregnation_method , Mesostructured, Nanomaterial	21
Reaction conditions	Temperature, Reaction time, H_2_ pressure	3
Structural parameters	Pore size, Surface area, Dispersion Mo, Slab length, Grade stacking	5
Other	Selectivity, Conversion of DBT	1

[a] Number of predictors used for regression.

Recently, the use of nanomaterials and mesostructured materials as supports has been widely explored to improve the performance of catalysts by modulating the surface area. Therefore, as mentioned above, relevant structural descriptors were incorporated, among them pore size, surface area, dispersion, slab length, and grade stacking. Some of these can correlate with the interaction between the catalyst and the support, which is an issue for HDS. However, as the characterization of these materials is challenging, there is limited structural information available. In total, 81 papers report the conversion of DBT, the selectivity, the reaction conditions, and the materials composition of derived catalysts from MoS_2_. Nevertheless, only 19.7 % of them were used to build the database because several articles do not report structural parameters, such as dispersion, stacking grade, and slab length. Out of these, in 19.7 % of articles, 86 different catalysts were identified.[^22^, ^23^, ^24^, ^25^, ^26^, ^27^, ^28^, ^29^, ^30^, ^31^, ^32^, ^33^, ^34^, ^35^, ^36^, ^37^, ^38^] The predictors here used can be classified into different categories: numeric and text data. The first has structural information and reaction conditions; the second includes materials, composition, and precursors. To describe the catalysts, supports, mesostructures, and nanomaterials, the predictors and their corresponding classes presented in Table 2 were considered.^[39]^

**Table 2:** Predictors associated with the composition of HDS catalyst. The classes for each predictor are shown in the supporting information.

Predictor	Description	Classes
Kind_catalyst	Kind of catalyst	CoMo, CoMoW, MoHet, NiMo, NiMoW
Promoters	Promoters in the catalyst	Co, Ni, None
No_Metals	Metals number in the catalyst	1, 2, 3
$Metal\_{cat1}^{\left[a\right]}$	First metal in the catalyst	Co, Ni, W, None
$Metal\_{cat2}^{\left[a\right]}$	Second metal in the catalyst	Co, Ni, None
P_Mo	Molybdenum precursor	(NH_4_)_6_Mo_7_O_24_, HPA_Mo, MoOx
P_Ni	Nickel precursor	Ni(NO_3_)_2_, NiSO_4_, None
P_Co	Cobalt precursor	Co(NO_3_)_2_, CoCO_3_, Co_organic, HPA_Co, None
P_W	Tungsten precursor	(NH_4_)_6_H_2_W_12_O_40_, None
Support_1	Support of the highest proportion	Al_2_O_3_, MCM, SBA, SiO, TUD, TiO_2_, zeolite, None
Support_2	Support of the lowest proportion	Al_2_O_3_, FDU, MO, SBA, TiO_2_, None
Element_1	Element of the highest proportion in the support	Al, Si, Ti, None
Element_2	Element in the support	Al, Si, Ti, Zr, Other_metal, None
Element_3	Element of the lowest proportion in the support	Ti, None
P_Si	Silicon precursor	SiO_2_, TEOS_Si, other, None
P_Al	Aluminum precursor	Al_2_O_3_, Al_inorganic, Al_organic, Bohemite_Al, None
P_Ti	Titanium precursor	Ti(i‐PrO)_4_, TiO_2_, None
Structure_directing_agent	Template polymers use for generating mesostructures	CTAB, F127, P123, TEG, None
Impregnation_method	Impregnation method of catalyst in the support	Coimpregnation, Successive, Other
Mesostructures	Mesostructured materials	Yes, No
Nanomaterial	Nanomaterial	Yes, No

[a] Without considering molybdenum.

The composition predictors were transformed into nominal values. The frequency plots of these nominal values are shown in the SI (Figure S1, Figure S2, and Figure S3). Also, Tables 3 and 4 show the descriptive statistics for numerical predictors. The more frequent reaction conditions are a temperature of 300 °C, 7.30 MPa of H_2_ pressure, and eight hours for time reaction. The mode of the data associated to the surface area predictor is of 182 m^2^/g, even though 75 percent of these data have higher values, which is indicative of a tendency to increase the surface area to get a better conversion percentage. Frequently, the stacking grade is associated with the interaction between the catalyst and the support, which should be balanced for optimal results. The mode of the data associated with the stacking grade is 2.4, a value close to the midpoint of such distribution, indicating that intermediate stacking grades are more common. Thus, staking could be a way to regulate the interaction between the catalyst and support, to improve the performance of these materials in HDS. The descriptive statistics for selectivity and conversion of DBT are shown in Table 5, where an outlier of selectivity with a value of 6.5 was replaced for the maximum selectivity without outliers. The reaction conditions are discrete values, whereas structural properties are continuous.

**Table 3:** Descriptive statistics for reaction conditions of HDS of DBT.

	Temperature (°C)	Pressure_H_2_ (MPa)	Reaction_time (h)
mean	302.62	5.20	7.34
std	21.01	2.22	2.05
min	275.00	0.10	2.00
25 %	300.00	3.00	6.00
50 %	300.00	6.00	8.00
75 %	300.00	7.30	8.00
max	350.00	7.30	10.00
mode	300.00	7.30	8.00

**Table 4:** Descriptive statistics for structural parameters of catalysts of HDS.

	Surface_area (m^2^/g)	Pore_size (nm)	Slab_length (Å)	Stacking grade	Dispersion
mean	336.95	7.22	43.56	2.59	0.29
std	160.11	3.43	20.65	0.89	0.09
min	17.10	0.50	5.23	1.10	0.11
25 %	200.50	5.05	32.93	1.92	0.24
50 %	322.00	6.31	39.40	2.50	0.29
75 %	479.65	7.60	45.60	3.20	0.34
max	662.00	17.90	113.00	5.70	0.70
mode	182.00	7.60	43.00	2.40	0.29

**Table 5:** Descriptive statistics for target variables.

	Selectivity	Conversion_DBT
mean	0.88	83.38
std	0.66	23.25
min	0.06	10.90
25 %	0.34	81.25
50 %	0.71	93.00
75 %	1.24	97.57
max	2.6	100.00
mode	1.21	94

It is worth noting that most of the conversion of DBT data are values above 80, which could result from the fact that usually only high yields and successful experiments are reported. Consequently, the information is limited for low‐value conversion.

All data were standardized; hence the minimum value of each variable is equal to 0, and the maximum is 1; this procedure is presented in the SI. Therefore, 30 predictors, of which 21 are related to composition, 3 to the experimental conditions, and 5 to structural parameters (Table 1), were considered. The last predictor for the estimation of conversion was the selectivity, and contrariwise, the conversion of DBT was used for the selectivity estimation.

In the present contribution, Random Forest, Lasso, and Ridge regression were employed for estimating the conversion of DBT and selectivity HYD/DDS. These methods have been widely used for prediction in datasets with limited data and a representative number of descriptors.[^17^, ^19^, ^20^] Recalling that a linear regression predicts the output variable ($\hat{Y}$ ), given the vector of regressor variables ($X_{j}$ ) as:

(1) $$\hat{Y}=\beta_{0}+\sum_{j=1}^{p}X_{j}{\hat{\beta }}_{j}$$

Where $\beta_{0}\;$ is the intercept term, ${\hat{\beta }}_{j}$ is the coefficient vector, and p is the number of predictors. Unlike the least squares classical method, which determines these coefficients by minimizing the residual sum of squares (RSS), regularized regressions include a regularized term to reduce overfitting and model complexity:[^40^, ^41^]

(2) $${\hat{\beta }}_{Ridge}={argmin}_{\beta }\left[\sum_{i=1}^{n}{\left(y_{i}-\beta_{0}\sum_{j=1}^{p}\beta_{j}x_{ij}\right)}^{2}+\lambda_{1}\sum_{j=1}^{p}\beta_{j}^{2}\right]$$

(3) $${\hat{\beta }}_{Lasso}={argmin}_{\beta }\left[\sum_{i=1}^{n}{\left(y_{i}-\beta_{0}\sum_{j=1}^{p}\beta_{j}x_{ij}\right)}^{2}+\lambda_{2}\sum_{j=1}^{p}\left|\beta_{j}\right|\right]$$

Where $\lambda_{1}$ and $\lambda_{2}$ correspond to hyperparameters; $y_{i}$ is the target variable, which can be the conversion of DBT or the selectivity (HYD/DDS); $x_{ij}$ are the values of the predictors. Ridge regression (eq. 2) contains an L2 norm as a penalty corresponding to the second term of equation 2. This helps to overcome multicollinearity as the penalization decreases significant coefficients without reaching zero. On the other hand, the Lasso method includes the L1 penalization, which is the second term of equation 3. It is worth mentioning that Ridge and Lasso algorithms carried out a cross‐validation, and in each iteration, the weights associated with the attributes involved in the regression are adjusted. This adjustment of weights avoids problems of overfitting and underfitting in the model. In this way, the iterations in Lasso cause there to be variables whose weights become zero. Therefore, only the attributes that correlate with the desired variable to be obtained in the linear regression remain. From the chemical point of view this would indicate with which attributes the dependent variable has a correlation. Random Forest is another method widely used to reduce overfitting, to increase prediction accuracy, and to get the importance of predictors. This is due to the Random Forest algorithm that operates as an ensemble method, combining the outcomes of multiple decision trees to mitigate overfitting and enhance the predictions in small sample datasets. Each tree is trained on distinct subsets of the original data using the Bootstrap technique. The random forest method identifies the relevant input features to estimate output features, providing outcomes similar to regularization methods for estimating conversion of DBT and selectivity. The Random Forest characteristics are advantageous for using in high‐dimensional datasets and various data distributions.^[17]^

Overfitting could be a relevant issue when using ML techniques with small amounts of data. However, the analyzed ML techniques deal with this issue using an implicit feature selection method. Moreover, the regularized term included in Lasso and Ridge methods is associated with a hyperparameter (lambda) to reduce the bias‐variance tradeoff.^[42]^ For these reasons, these hyperparameters were optimized by a 10‐fold cross‐validations strategy, which resamples and splits the dataset into ten groups, one of which is used as a test set and the rest is the train set; this procedure is iterated ten times.^[15]^ Three metrics to evaluate the performance and quality of regression methods were used. The first is the determination coefficient $(R^{2}$ ), with a range score from 0 to 1. A value equal to 1 corresponds to the perfect performance. This metric is calculated as the squared Pearson's correlation coefficient. The predictions deviation of each model was measured through the mean absolute error (MAE) and the root mean square error (RMSE):

(4) $$MAE=\frac{1}{N}\sum_{i=1}^{N}\left|Y_{i}-Y_{i}^{pred}\right|$$

(5) $$RMSE=\frac{1}{N}\sqrt{\sum_{i=1}^{N}{\left(Y_{i}-Y_{i}^{pred}\right)}^{2}}\;$$

Where $Y_{i}$ corresponds to an observed variable target and $Y_{i}^{pred}$ to a predicted variable.^[17]^ The metrics reported $R^{2}$ , MAE, and RMSE correspond to the mean obtained by ten‐fold cross‐validations. The implementation of methods, hyperparameters optimization, and analyses were performed in R‐studio and are described in the supporting information.

## Results and Discussion

The estimation of the conversion of DBT by different methods exhibits interesting aspects. As shown in Table 6, the Ridge regression offers the best $R^{2}$ (0.91) although a high RMSE and MAE (0.2594 and 0.1841, respectively). This fact implies that its predictions are deficient compared to the other methods. The Lasso method has RMSE and MAE values (0.0809 and 0.0623, respectively) lower than the ones obtained with Ridge, i. e. the Lasso method has a better predictive capability. Both regularized regressions exhibit significant differences between their metrics, which could be due to the selection of predictors of the Lasso method since it only selects 23 out of 30.

**Table 6:** Metrics values for employed regression methods in the prediction of conversion of DBT.

Method	$R^{2}$	RMSE	MAE
Ridge	0.91	0.2594	0.1841
Lasso	0.90	0.0809	0.0623
Random Forest	0.79	0.1031	0.0737

**Figure 1:**
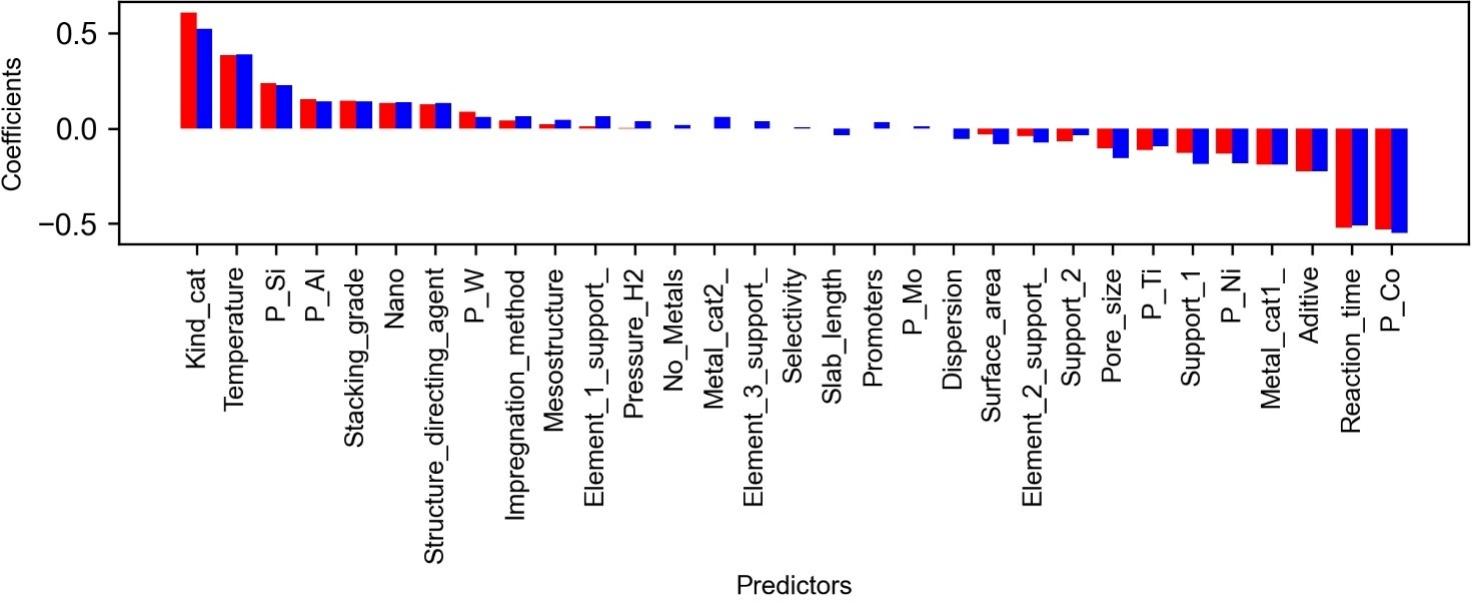
300 Coefficients of Lasso and Ridge regressions for estimation of conversion of DBT. The numerical values of these regression coefficients are shown in Table S2; the value of $\beta_{0}^{Ridge}$ is 1.15, and $\beta_{0}^{Lasso}$ is 1.14.

The regression coefficients exhibit the effect of the predictors on the target variable.^[43]^ The predictors with the highest value coefficients are the kind of catalyst (Kind_cat ), the cobalt precursors (P_Co ), reaction time, and temperature (Figure 1). The first two predictors mentioned and the other three, such as Additive, Metal_cat1 , and P_Ni , describe the composition of the catalysts in an independent way from the support. On the other hand, the silicon and aluminum precursors (P_Si and P_Al ) represent the support composition. The structural description and the interaction between catalyst and support could be poorly considered because Lasso only selects three structural properties, and their corresponding coefficients are low; the stacking grade is the most representative. It is worth noting, that the surface area has a coefficient near zero, i. e. it is little relevant to the model. In contrast, it has been suggested that the surface area is narrowly associated with the performance of the HDS catalyst.[^4^, ^12^] These aspects could be limitations for estimating the HDS conversion of DBT.

**Figure 2:**
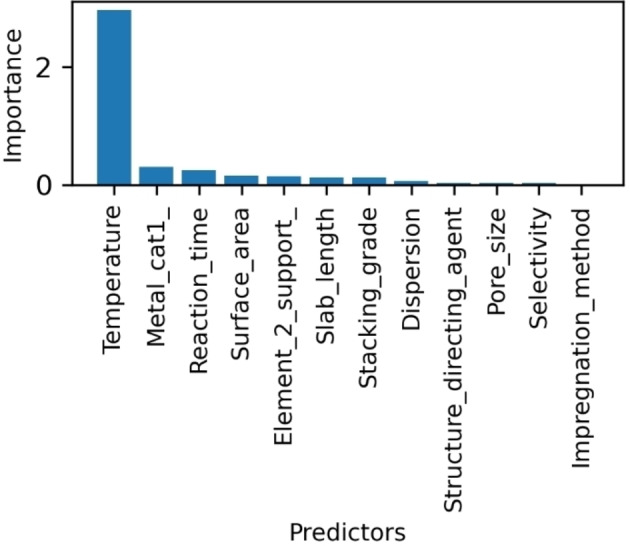
Importance of relevant predictors for the estimation of conversion of DBT by Random Forest regression.

An alternative to the regularized regressions is the Random Forest method, which offers some advantages, as it has low sensitivity to preprocessing and is easily tunable. Although random forest lacks the interpretability of the decision trees method, the model can be analyzed through the importance of predictors. For the estimation of the conversion of DBT by random forest, the determination coefficient $R^{2}\;$ is 0.79 and it is lower than the obtained by the regularized regressions. However, $R^{2}$ still denotes a viable model, and the predictors are adequate for estimating the HDS conversion of DBT. On the other hand, MAE and RMSE values are 0.0737 and 0.1031, respectively, representing the lowest error in the prediction and a significant adjustment. As shown in Figure 2, the most representative predictor is temperature. Moreover, other significant predictors describe the catalyst composition and structural properties. Among these predictors, it is worth mentioning the selectivity that is related to HDS reaction mechanism. As mentioned above, the surface area has been associated with the performance and efficiency of the HDS catalysts, which agrees with the obtained importance of this property. Notably, the structural properties, such as the slab length, grade stacking, and surface area, are contemplated in the Random Forest model. These predictors could be associated with the interaction between the catalyst and support that have a relevant effect on the HDS.[^11^, ^12^] However, the predictors of high importance do not agree with those selected through the Lasso method. In this way, a data mining study focusing on identifying the relevant patterns associated with the conversion of DBT could be appropriate in this context. However, this analysis is out of the scope of this contribution.

**Figure 3:**
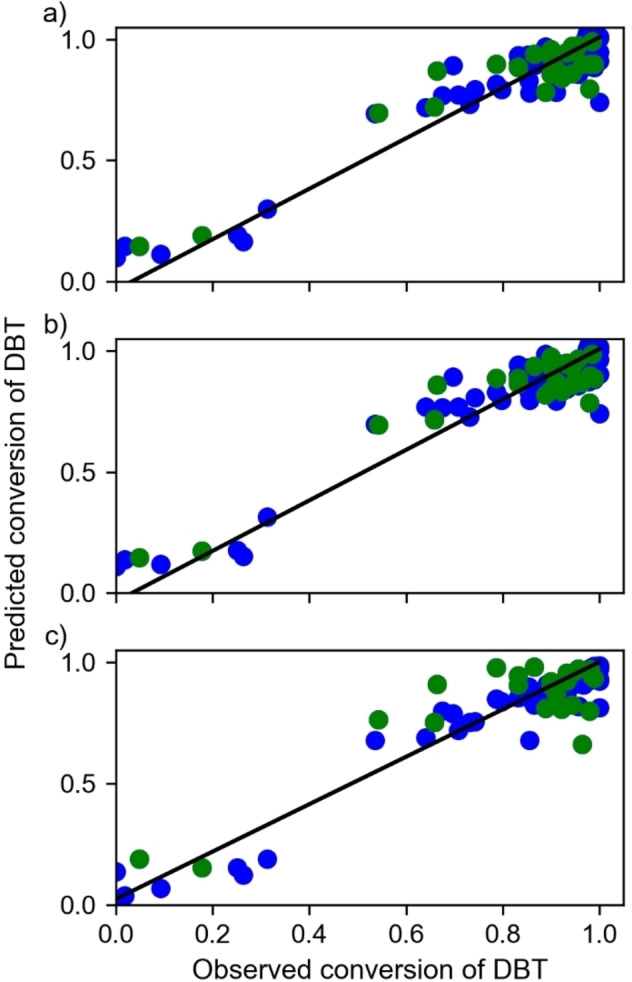
Dispersion plots for observed conversion of DBT versus predicted conversion of DBT by a) Ridge regression, b) Lasso regression, and c) Random Forest regression. The green dots correspond to the data train, and the blue dots are data tests.

For all regression methods, the predictions around the low conversions of DBT are more deficient than those corresponding to high values (Figure 3). This fact could result from the need for more data with low conversion, which is a consequence of the bias in scientific literature where only the results with a high yield are reported, and valuable information about the experiment with median and low performance is discarded. However, this loss of information is vital for training ML algorithms. In this way, J.M. Cole discussed the limitations of quantitative predictions through ML implementation associated with the synthetic design and the report of practice deficiencies, especially in the report of chemical low‐yielding, generating a high bias and affecting the predictions.^[14]^ Indeed, covering the chemical space is a challenge and crucial for getting appropriate predictions and interpretations. Despite the limitation above mentioned, the Random Forest model is used to estimate the conversion of DBT, which recovers essential chemical information that agrees with the experimental interpretation of the derived catalysts from MoS_2_.

The selectivity of HDS processes is an essential factor, closely related to the reaction mechanism, which can proceed through either the HYD or DDS pathways. Therefore, estimating selectivity can help to understand critical chemical issues and design efficient catalysts. Table 7 presents selectivity estimation metrics obtained through regression methods. It is important to notice that the MAE and RMSE of regularized regressions exhibit higher values than the ones of Random Forest. Thus, this last method has a deficient prediction quality compared with the other analyzed regressions.

**Table 7:** Metrics values for employed regression methods in the prediction of selectivity.

Method	$R^{2}$	RMSE	MAE
Ridge	0.84	0.0981	0.0756
Lasso	0.84	0.1098	0.0865
Random Forest	0.72	0.1406	0.1038

Regarding the estimation of selectivity through regularized regressions, both methods offer similar performance since their $R^{2}$ (0.84) is identical. The Ridge regression has the best predictions according to the metrics MAE and RMSE (0.0981 and 0.0756). These last metrics for Lasso regression are higher, therefore the Lasso prediction capability is slightly lower. However, the advantage of the Lasso regression is the selection of predictors, as it only considers twenty‐one predictors and offers a similar description to the one obtained with the Ridge method.

**Figure 4:**
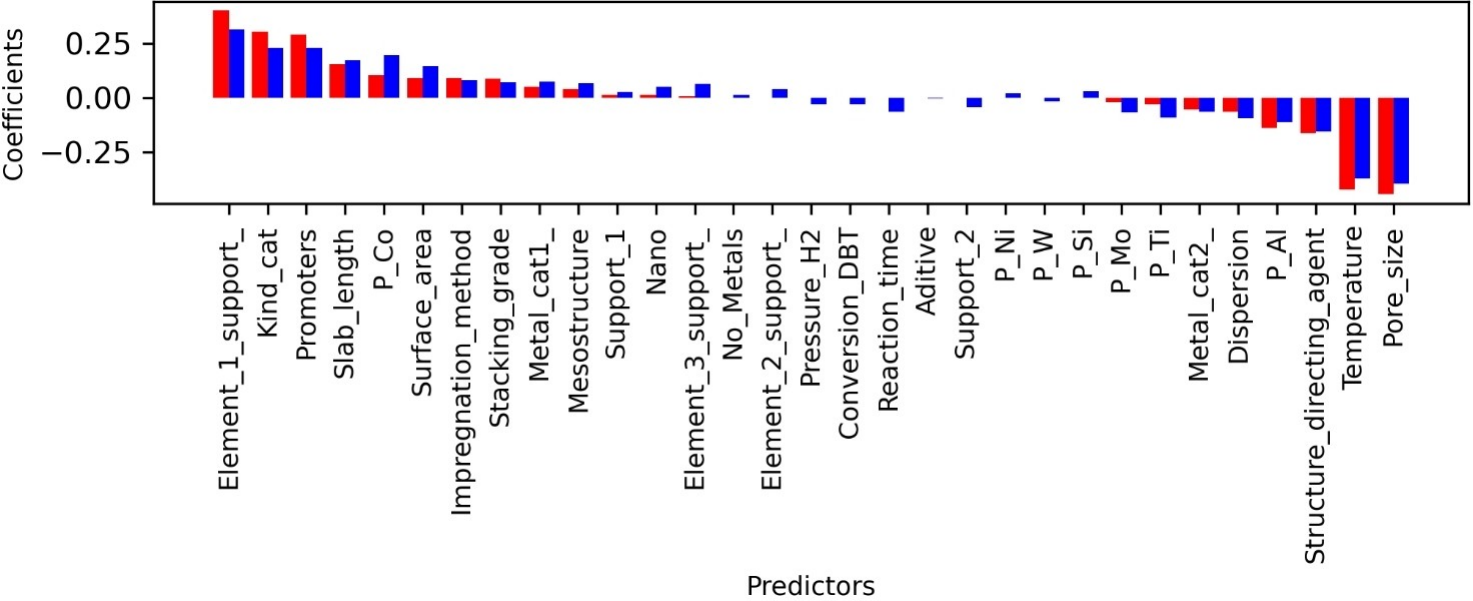
Coefficients of Lasso and Ridge regressions for estimation of selectivity. The numerical values of these regression coefficients are shown in Table S3; the value of $\beta_{0}^{Ridge}$ is 0.23, and $\beta_{0}^{Lasso}$ is 0.22.

On selectivity prediction, the magnitude of regression coefficients for Ridge and Lasso present similar tendencies (Figure 4). Considering these coefficients, the pore size is a crucial predictor, as well as other properties associated with the structure of the material, such as the staking grade, the slab length, and the surface area. It is worth noting that these descriptors collect data on the fundamental aspects of HDS reaction mechanisms, which at least describe vital issues. Firstly, the surface area is related to the availability of active sites and their possible accessibility for the reaction of DBT molecules on the edge of catalysts. Secondly, the staking grade could be associated with the interaction between support and catalyst, modulating the reactivity of active sites to promote the DDS or HYD. Lastly, the pore size is closely linked to their diffusion properties, influencing the catalyst performance. In this way, diffusion can considerably impact HDS processes, particularly those involving mesostructured materials.[^44^, ^45^] Another aspect that points to the importance of mesostructured supports is the high coefficient for structure‐directing agents, which are the compounds used for generating diverse morphologies in the supports. The outlook obtained through the coefficient analysis captures the connection of the key structural aspects for estimating selectivity, which agrees with the experimental interpretations.

**Figure 5:**
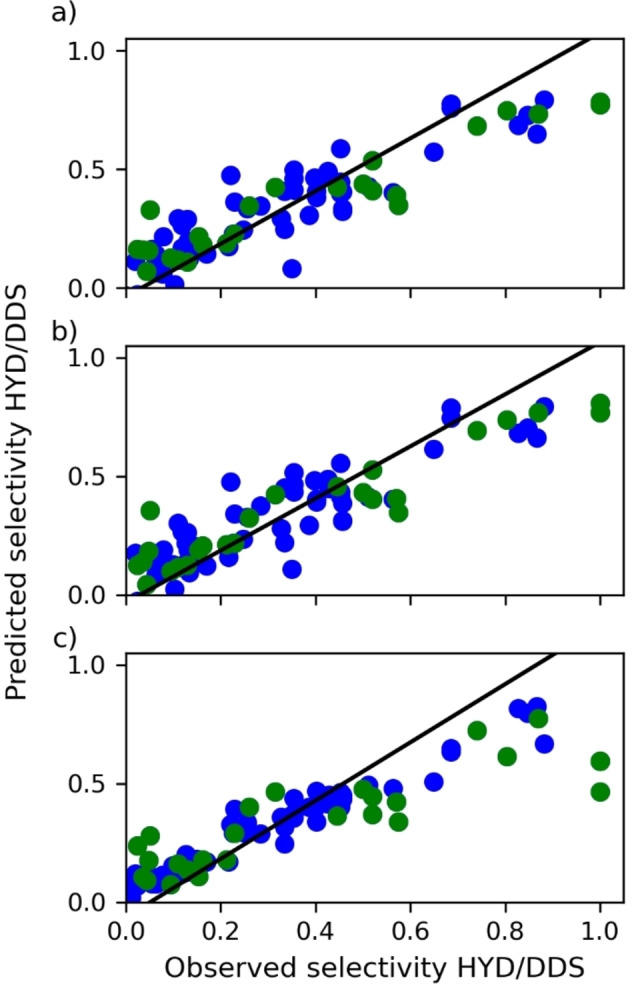
Dispersion plots for observed selectivity vs predicted selectivity by a) Ridge regression, b) Lasso regression, and c) Random Forest. The green dots correspond to the data train, and the blue dots are data test

As shown in Figure 5, the regularized regressions exhibit a similar relationship between observed and predicted selectivity, so both methods offer similar selectivity estimations. Although Random Forest regression has a poor adjustment in the selectivity prediction, the three regression methods have a similar pattern as underestimating selectivity to high values. This underestimating is more representative in the random forest than for regularized regressions, which limits its prediction capability.

The complexity of the chemical environment associated with HDS is a challenge for predicting conversion and selectivity. This is due to the consideration of five kinds of catalysts (NiMo, CoMo, MoHet, NiMoW, and CoMoW), in combination with diverse supports and additives, generating several chemical environments for active sites. However, the experimental characterization of these sites is difficult or limited. Therefore, one way to include this information is through experimental measurements of structural parameters such as pore size, molybdenum atom dispersion, and slab length, among others. The considered predictors are key points for the HDS of DBT, and the analyzed metrics confirm that the selectivity and conversion estimations are adequate. Another advantage of these methods is that they allow identifying predictors relevant to the estimation of target variables by analyzing regression coefficients. In this way, the kind of catalyst, the composition of support and the size of the pore, molybdenum atom dispersion, and slab length are identified as relevant to the estimation of selectivity. This description agrees with previous experimental reports.[^4^, ^12^] Finally, the achieved perspective about the HDS and the derived catalysts of MoS_2_ could support catalyst design and the optimization of this process.

## Conclusions

In this work, ML algorithms were used to analyze the performance of catalysts derived from MoS_2_ in HDS of DBT by estimating the conversion of DBT and selectivity. The evaluation of these targets by Lasso, Ridge, and Random Forest regressions allowed the identification of the most suitable method, and their analysis helped to recognize the advantages and limitations of each one. Furthermore, Ridge and Lasso regressions are highlighted as useful tools for datasets with limited data.

The most deficient method for estimating the conversion of DBT is Ridge regression. At the same time, Random Forest has an adequate capacity for conversion prediction and considers selectivity and surface area as relevant predictors, which agrees with the corresponding scientific reports.

On the other hand, regularized regressions are appropriate for determining selectivity. Analyzing regression coefficients supports the identification of crucial predictors for selectivity estimation, such as pore size, surface area, slab length, and stacking grade, which could be associated with the active sites and diffusion phenomena in these materials. The information provided offers crucial insight into the HDS catalysts and the key factors influencing their performance. This depiction coincides with the understanding derived from experimental observations.

## Supporting Information

Supporting information contains a document with the frequency plots, histograms of the dataset, and regression coefficients (PDF).

The dataset of HDS catalysts, the methods of regression, and the hyperparameter optimization implemented in R can be consulted in the GitHub repository at URL: https://github.com/lunamgcg/HDS_regression

## Conflict of Interests

The authors declare no conflict of interest.

1

## Supporting information

As a service to our authors and readers, this journal provides supporting information supplied by the authors. Such materials are peer reviewed and may be re‐organized for online delivery, but are not copy‐edited or typeset. Technical support issues arising from supporting information (other than missing files) should be addressed to the authors.

Supporting Information

## Data Availability

The data that support the findings of this study are available in the supplementary material of this article.
