# Supplementary material for: Hydrodesulfurization of Dibenzothiophene: A Machine Learning Approach
Source: ChemistryOpen. 2024 Apr 12;13(9):e202400062. doi: 10.1002/open.202400062 (PMC11633359; doi:10.1002/open.202400062)
Supplement: Supplementary file 1 — Supporting Information [file OPEN-13-e202400062-s001.pdf]

ChemistryOpen

Supporting Information

Hydrodesulfurization of Dibenzothiophene: A Machine Learning Approach

Guadalupe Castro,* Julián Cruz-Borbolla, Marcelo Galván, Alfredo Guevara-García, Joel Ireta, Myrna H. Matus, Amilcar Meneses-Viveros, Luis Ignacio Perea-Ramírez, and Miriam Pescador-Rojas

Supporting Information for:

Hydrodesulfurization of dibenzothiophene: a machine learning approach

Guadalupe Castro,*[a] Julián Cruz-Borbolla,[b] Marcelo Galván, [a] Alfredo Guevara-García,[c] Joel Ireta, [a] Myrna H. Matus, [d] Amilcar Meneses-Viveros, [e] Luis I. Perea-Ramírez, [d] Miriam Pescador-Rojas[f]

[a] Dr. Guadalupe Castro
Departamento de Química
Universidad Autónoma Metropolitana-Iztapalapa
Av. Ferrocarril San Rafael Atlixco 186, Col. Leyes de Reforma 1 A Sección, Iztapalapa, C.P. 09310, Ciudad de México, México
E-mail: castrog@xanum.uam.mx

[b] Dr. Julián Cruz-Borbolla
Área Académica de Química
Centro de Investigaciones Químicas - Universidad Autónoma del Estado de Hidalgo
Carretera Pachuca-Tulancingo km. 4.5, Ciudad del Conocimiento, C.P. 42184, Mineral de la Reforma, Hidalgo, México

[a] Dr. Marcelo Galván
Departamento de Química
Universidad Autónoma Metropolitana-Iztapalapa
Av. Ferrocarril San Rafael Atlixco 186, Col. Leyes de Reforma 1 A Sección, Iztapalapa, C.P. 09310, Ciudad de México, México

[c] Dr. Alfredo Guevara-García
Departamento de Química
CONAHCYT-Universidad Autónoma Metropolitana-Iztapalapa
Av. Ferrocarril San Rafael Atlixco 186, Col. Leyes de Reforma 1 A Sección, Iztapalapa, C.P. 09310, Ciudad de México, México

[a] Dr. Joel Ireta
Departamento de Química
Universidad Autónoma Metropolitana-Iztapalapa
Av. Ferrocarril San Rafael Atlixco 186, Col. Leyes de Reforma 1 A Sección, Iztapalapa, C.P. 09310, Ciudad de México, México

[d] Dr. Myrna H. Matus
Instituto de Química Aplicada
Universidad Veracruzana
Av. Luis Castelazo Ayala s/n, Col. Industrial-Ánimas, A.P. 575, Xalapa, Ver., México

[e] Dr. Amilcar Meneses-Viveros
Departamento de Computación
CINVESTAV-IPN
Av. IPN 2508, Col. San Pedro Zacatenco, C.P. 07360, Ciudad de Mexico, México

[d] Dr. Luis I. Perea-Ramírez
Instituto de Química Aplicada
Universidad Veracruzana
Av. Luis Castelazo Ayala s/n, Col. Industrial-Ánimas, A.P. 575, Xalapa, Ver., México

[f] Miriam Pescador-Rojas
Escuela Superior de Cómputo, Instituto Politécnico Nacional
Instituto Politécnico Nacional
Av. Juan de Dios Bátiz s/n, esq. Av. Miguel Othón de Mendizabal, Col. Lindavista, Gustavo A. Madero, C. P. 07738, Ciudad de México, México

.

Data collection.

The data was collected by searching the scientific databases of Scopus, Web of Science, and SciFinder for keywords such as desulfurization, hydrodesulfurization, deep desulfurization, catalysis, heavy crude oil, and petroleum; considering a publication period from 2005 until 2022. Files (CSV) with information about articles in an ordered format were obtained from these databases. Only research articles about the HDS of DBT promoted by new catalysts were taken into account. The conference reports, reviews, and books were not included because these publications omit relevant information about the catalyst. Data extraction was carried out through data capture from articles in format pdf.
To select articles containing target variables, the keywords DBT, catalyst, conversion, and selectivity were searched for in the abstract. Afterward, the predictors were defined to consider essential information describing the HDS process, such as composition, structural parameters of catalyst, and reaction condition. The composition predictors were determined by classes of atoms included in the catalyst and support and kind mesostructure correspondent. The structural parameters look forward to indirectly describing the catalytic sites, so pore size, surface area, molybdenum atom dispersion, slab length, and grade stacking were chosen.
In the second stage, only articles that report the target variables and predictors selected were considered to obtain a complete dataset without missing data. For each article, all catalysts for HDS of DBT and their properties were compiled.

Table S1.Catalysts included in the data set and their sources.

No. Material	catalyst	doi	Source
1	Co-PMo/Al2O3	10.1016/j.cattod.2020.10.010	Glotov, A.P.; Vutolkina, A.V.; Vinogradov, N.A.; Pimerzin, A.A.; Vinokurov, V.A.; Pimerzin, A. Enhanced HDS and HYD activity of sulfide Co-PMo catalyst supported on alumina and structured mesoporous silica composite, Catalysis Today, 2021, 377, 82–91.
2	Co-PMo/MCM-41-Al2O3	10.1016/j.cattod.2020.10.010	Glotov, A.P.; Vutolkina, A.V.; Vinogradov, N.A.; Pimerzin, A.A.; Vinokurov, V.A.; Pimerzin, A. Enhanced HDS and HYD activity of sulfide Co-PMo catalyst supported on alumina and structured mesoporous silica composite, Catalysis Today, 2021, 377, 82–91.
3	CMW/SBA	10.1016/j.cattod.2019.06.023	Mendoza-Nieto, J. A; Vizueth-Montes de Oca, A.; Calzada, L. A.; Klimova, T. E. Trimetallic NiMoW and CoMoW catalysts supported on SBA-15 modified with titania or zirconia for deep hydrodesulfurization, Catalysis Today, 2021, 360, 78–89.
4	CMW/Zr-SBA	10.1016/j.cattod.2019.06.023	Mendoza-Nieto, J. A; Vizueth-Montes de Oca, A.; Calzada, L. A.; Klimova, T. E. Trimetallic NiMoW and CoMoW catalysts supported on SBA-15 modified with titania or zirconia for deep hydrodesulfurization, Catalysis Today, 2021, 360, 78–89.
5	CMW/Ti-SBA	10.1016/j.cattod.2019.06.023	Mendoza-Nieto, J. A; Vizueth-Montes de Oca, A.; Calzada, L. A.; Klimova, T. E. Trimetallic NiMoW and CoMoW catalysts supported on SBA-15 modified with titania or zirconia for deep hydrodesulfurization, Catalysis Today, 2021, 360, 78–

			89.
6	NMW/SBA	10.1016/j.cattod.2019.06.023	Mendoza-Nieto, J. A; Vizueth-Montes de Oca, A.; Calzada, L. A.; Klimova, T. E. Trimetallic NiMoW and CoMoW catalysts supported on SBA-15 modified with titania or zirconia for deep hydrodesulfurization, Catalysis Today, 2021, 360, 78–89.
7	NMW/Zr-SBA	10.1016/j.cattod.2019.06.023	Mendoza-Nieto, J. A; Vizueth-Montes de Oca, A.; Calzada, L. A.; Klimova, T. E. Trimetallic NiMoW and CoMoW catalysts supported on SBA-15 modified with titania or zirconia for deep hydrodesulfurization, Catalysis Today, 2021, 360, 78–89.
8	NMW/Ti-SBA	10.1016/j.cattod.2019.06.023	Mendoza-Nieto, J. A; Vizueth-Montes de Oca, A.; Calzada, L. A.; Klimova, T. E. Trimetallic NiMoW and CoMoW catalysts supported on SBA-15 modified with titania or zirconia for deep hydrodesulfurization, Catalysis Today, 2021, 360, 78–89.
9	NiMo/TiO2	10.1007/s11244-020-01253-8	Castillo, L. J. R.; Alarcon, L. E.; Klimova, T. E. Exotic Nanostructured Titania Supports for Deep Hydrodesulfurization Catalysts: Are They Better Than the Conventional Ones?, Topics in Catalysis 2020, 63, 511–528.
10	NiMo/NT72	10.1007/s11244-020-01253-8	Castillo, L. J. R.; Alarcon, L. E.; Klimova, T. E. Exotic Nanostructured Titania Supports for Deep Hydrodesulfurization Catalysts: Are They Better Than the Conventional Ones?, Topics in Catalysis 2020, 63, 511–528.
11	NiMo/gamma-Al2O3	10.1007/s11244-020-01253-8	Castillo, L. J. R.; Alarcon, L. E.; Klimova, T. E. Exotic Nanostructured Titania Supports for Deep Hydrodesulfurization Catalysts: Are They Better Than the Conventional Ones?, Topics in Catalysis 2020, 63, 511–528.
12	NiMo/NT24	10.1007/s11244-020-01253-8	Castillo, L. J. R.; Alarcon, L. E.; Klimova, T. E. Exotic Nanostructured Titania Supports for Deep Hydrodesulfurization Catalysts: Are They Better Than the Conventional Ones?, Topics in Catalysis 2020, 63, 511–528.
13	NiMo/NT	10.1007/s11244-020-01253-8	Castillo, L. J. R.; Alarcon, L. E.; Klimova, T. E. Exotic Nanostructured Titania Supports for Deep Hydrodesulfurization Catalysts: Are They Better Than the Conventional Ones?, Topics in Catalysis 2020, 63, 511–528.
14	CAT-Sulf	10.1039/d0cy01004a	Liu, Z. W.; Han, W.; Hu, D. W.; Nie, H.; Wang, Z.; Sun, S. L.; Deng, Z. H.; Yang, Q.H. Promoting effects of SO42- on a NiMo/gamma-Al2O3 hydrodesulfurization catalyst, Catal. Sci. Technol. 2020,10, 5218?5230.
15	CAT-Nit	10.1039/d0cy01004a	Liu, Z. W.; Han, W.; Hu, D. W.; Nie, H.; Wang, Z.; Sun, S. L.; Deng, Z. H.; Yang, Q.H. Promoting effects of SO42- on a NiMo/gamma-Al2O3 hydrodesulfurization catalyst, Catal. Sci. Technol. 2020,10, 5218?5230.
16	CAT-NiP	10.1039/d0cy01004a	Liu, Z. W.; Han, W.; Hu, D. W.; Nie, H.; Wang, Z.; Sun, S. L.; Deng, Z. H.; Yang, Q.H. Promoting effects of SO42- on a NiMo/gamma-Al2O3 hydrodesulfurization catalyst, Catal. Sci. Technol. 2020,10, 5218?5230.
17	NiMoZrM	10.1016/j.cattod.2018.03.039	Mendez, F. J.; Bravo-Ascencion, G.; Gonzalez-Mota, M.; Puente-Lee, I.; Bokhimi, X.; Klimova, T. E. NiMo catalysts supported on Al, Nb, Ti or Zr-containing MCM-41 for dibenzothiophene hydrodesulfurization, Catalysis Today 2020, 349, 217–227.
18	NiMoTiM	10.1016/j.cattod.2018.03.039	Mendez, F. J.; Bravo-Ascencion, G.; Gonzalez-Mota, M.; Puente-Lee, I.; Bokhimi, X.; Klimova, T. E. NiMo catalysts supported on Al, Nb, Ti or Zr-containing MCM-41 for dibenzothiophene hydrodesulfurization, Catalysis Today 2020, 349, 217–227.

19	NiMoAlM	10.1016/j.cattod.2018.03.039	Mendez, F. J.; Bravo-Ascencion, G.; Gonzalez-Mota, M.; Puente-Lee, I.; Bokhimi, X.; Klimova, T. E. NiMo catalysts supported on Al, Nb, Ti or Zr-containing MCM-41 for dibenzothiophene hydrodesulfurization, Catalysis Today 2020, 349, 217–227.
20	NiMoSiM	10.1016/j.cattod.2018.03.039	Mendez, F. J.; Bravo-Ascencion, G.; Gonzalez-Mota, M.; Puente-Lee, I.; Bokhimi, X.; Klimova, T. E. NiMo catalysts supported on Al, Nb, Ti or Zr-containing MCM-41 for dibenzothiophene hydrodesulfurization, Catalysis Today 2020, 349, 217–227.
21	NiMoNbM	10.1016/j.cattod.2018.03.039	Mendez, F. J.; Bravo-Ascencion, G.; Gonzalez-Mota, M.; Puente-Lee, I.; Bokhimi, X.; Klimova, T. E. NiMo catalysts supported on Al, Nb, Ti or Zr-containing MCM-41 for dibenzothiophene hydrodesulfurization, Catalysis Today 2020, 349, 217–227.
22	Z5-NiMo-10h	10.1016/j.apcata.2019.117113	Dong, C. W.; Yin, C. L.; Wu, T. T.; Wu, Z. Y.; Liu, D.; Liu, C. G. Study on the modification of unsupported hydrodesulfurization catalysts by the ZSM-5 zeolite nanoclusters, Applied Catalysis A, General 2019, 582, 117113
23	Z5-NiMo-30g	10.1016/j.apcata.2019.117113	Dong, C. W.; Yin, C. L.; Wu, T. T.; Wu, Z. Y.; Liu, D.; Liu, C. G. Study on the modification of unsupported hydrodesulfurization catalysts by the ZSM-5 zeolite nanoclusters, Applied Catalysis A, General 2019, 582, 117114
24	Z5-NiMo-20g	10.1016/j.apcata.2019.117113	Dong, C. W.; Yin, C. L.; Wu, T. T.; Wu, Z. Y.; Liu, D.; Liu, C. G. Study on the modification of unsupported hydrodesulfurization catalysts by the ZSM-5 zeolite nanoclusters, Applied Catalysis A, General 2019, 582, 117115
25	Z5-NiMo-24h	10.1016/j.apcata.2019.117113	Dong, C. W.; Yin, C. L.; Wu, T. T.; Wu, Z. Y.; Liu, D.; Liu, C. G. Study on the modification of unsupported hydrodesulfurization catalysts by the ZSM-5 zeolite nanoclusters, Applied Catalysis A, General 2019, 582, 117116
26	Z5-NiMo-10g	10.1016/j.apcata.2019.117113	Dong, C. W.; Yin, C. L.; Wu, T. T.; Wu, Z. Y.; Liu, D.; Liu, C. G. Study on the modification of unsupported hydrodesulfurization catalysts by the ZSM-5 zeolite nanoclusters, Applied Catalysis A, General 2019, 582, 117117
27	Z5-NiMo-14h	10.1016/j.apcata.2019.117113	Dong, C. W.; Yin, C. L.; Wu, T. T.; Wu, Z. Y.; Liu, D.; Liu, C. G. Study on the modification of unsupported hydrodesulfurization catalysts by the ZSM-5 zeolite nanoclusters, Applied Catalysis A, General 2019, 582, 117118
28	Z5-NiMo18h	10.1016/j.apcata.2019.117113	Dong, C. W.; Yin, C. L.; Wu, T. T.; Wu, Z. Y.; Liu, D.; Liu, C. G. Study on the modification of unsupported hydrodesulfurization catalysts by the ZSM-5 zeolite nanoclusters, Applied Catalysis A, General 2019, 582, 117119
29	4-Mo/Al2O3	10.1007/s10562-018-2480-7	Kokliukhin, A.; Nikulshina, M.; Sheldaisov-Meshcheryakov, A.; Mozhaev, A.; Nikulshin, P., CoMo Hydrotreating Catalysts Supported on Al2O3, SiO2 and SBA-15 Prepared from Single Co2Mo10-Heteropolyacid: In Search of Self-Promotion Effect, Catalysis Letters, 2018, 148, 2869–2879.
30	2-CoMo/Al2O3	10.1007/s10562-018-2480-7	Kokliukhin, A.; Nikulshina, M.; Sheldaisov-Meshcheryakov, A.; Mozhaev, A.; Nikulshin, P., CoMo Hydrotreating Catalysts Supported on Al2O3, SiO2 and SBA-15 Prepared from Single Co2Mo10-Heteropolyacid: In Search of Self-Promotion Effect, Catalysis Letters, 2018, 148, 2869–2879.
31	4-CoMo/Al2O3	10.1007/s10562-018-2480-7	Kokliukhin, A.; Nikulshina, M.; Sheldaisov-Meshcheryakov, A.; Mozhaev, A.; Nikulshin, P., CoMo Hydrotreating Catalysts Supported on Al2O3, SiO2 and SBA-15 Prepared from Single Co2Mo10-Heteropolyacid: In Search of Self-Promotion Effect, Catalysis Letters, 2018, 148, 2869–2879.
32	1Mo/SBA-15	10.1007/s10562-018-2480-7	Kokliukhin, A.; Nikulshina, M.; Sheldaisov-Meshcheryakov, A.; Mozhaev, A.;

			Nikulshin, P., CoMo Hydrotreating Catalysts Supported on Al2O3, SiO2 and SBA-15 Prepared from Single Co2Mo10-Heteropolyacid: In Search of Self-Promotion Effect, Catalysis Letters, 2018, 148, 2869–2879.
33	1-CoMo/SBA-15	10.1007/s10562-018-2480-7	Kokliukhin, A.; Nikulshina, M.; Sheldaisov-Meshcheryakov, A.; Mozhaev, A.; Nikulshin, P., CoMo Hydrotreating Catalysts Supported on Al2O3, SiO2 and SBA-15 Prepared from Single Co2Mo10-Heteropolyacid: In Search of Self-Promotion Effect, Catalysis Letters, 2018, 148, 2869–2879.
34	2-CoMo/SBA-15	10.1007/s10562-018-2480-7	Kokliukhin, A.; Nikulshina, M.; Sheldaisov-Meshcheryakov, A.; Mozhaev, A.; Nikulshin, P., CoMo Hydrotreating Catalysts Supported on Al2O3, SiO2 and SBA-15 Prepared from Single Co2Mo10-Heteropolyacid: In Search of Self-Promotion Effect, Catalysis Letters, 2018, 148, 2869–2879.
35	4-CoMo/SBA-15	10.1007/s10562-018-2480-7	Kokliukhin, A.; Nikulshina, M.; Sheldaisov-Meshcheryakov, A.; Mozhaev, A.; Nikulshin, P., CoMo Hydrotreating Catalysts Supported on Al2O3, SiO2 and SBA-15 Prepared from Single Co2Mo10-Heteropolyacid: In Search of Self-Promotion Effect, Catalysis Letters, 2018, 148, 2869–2879.
36	1-CoMo/SiO2	10.1007/s10562-018-2480-7	Kokliukhin, A.; Nikulshina, M.; Sheldaisov-Meshcheryakov, A.; Mozhaev, A.; Nikulshin, P., CoMo Hydrotreating Catalysts Supported on Al2O3, SiO2 and SBA-15 Prepared from Single Co2Mo10-Heteropolyacid: In Search of Self-Promotion Effect, Catalysis Letters, 2018, 148, 2869–2879.
37	4-CoMo/SiO2	10.1007/s10562-018-2480-7	Kokliukhin, A.; Nikulshina, M.; Sheldaisov-Meshcheryakov, A.; Mozhaev, A.; Nikulshin, P., CoMo Hydrotreating Catalysts Supported on Al2O3, SiO2 and SBA-15 Prepared from Single Co2Mo10-Heteropolyacid: In Search of Self-Promotion Effect, Catalysis Letters, 2018, 148, 2869–2879.
38	NiMo/ZF-80	10.1016/j.fuproc.2018.08.010	Li, Y. Y.; Chi, K. B.; Zhang, H. L.; Du, P.; Hu, D.; Xiao, C. K.; Li, H. P.; Zhao, Z.; Duan, A. J.; Xu, C. M. The influence of hydrothermal crystallization temperature on a novel FDU-12 mesoporous composite assembled by ZSM-5 nanoclusters and its hydrodesulfurization performance for DBT and FCC diesel, Fuel Processing Technology 2018, 180, 56–66.
39	NiMo/ZF-100	10.1016/j.fuproc.2018.08.010	Li, Y. Y.; Chi, K. B.; Zhang, H. L.; Du, P.; Hu, D.; Xiao, C. K.; Li, H. P.; Zhao, Z.; Duan, A. J.; Xu, C. M. The influence of hydrothermal crystallization temperature on a novel FDU-12 mesoporous composite assembled by ZSM-5 nanoclusters and its hydrodesulfurization performance for DBT and FCC diesel, Fuel Processing Technology 2018, 180, 56–66.
40	NiMo/ZF-110	10.1016/j.fuproc.2018.08.010	Li, Y. Y.; Chi, K. B.; Zhang, H. L.; Du, P.; Hu, D.; Xiao, C. K.; Li, H. P.; Zhao, Z.; Duan, A. J.; Xu, C. M. The influence of hydrothermal crystallization temperature on a novel FDU-12 mesoporous composite assembled by ZSM-5 nanoclusters and its hydrodesulfurization performance for DBT and FCC diesel, Fuel Processing Technology 2018, 180, 56–66.
41	NiMo/ZF-120	10.1016/j.fuproc.2018.08.010	Li, Y. Y.; Chi, K. B.; Zhang, H. L.; Du, P.; Hu, D.; Xiao, C. K.; Li, H. P.; Zhao, Z.; Duan, A. J.; Xu, C. M. The influence of hydrothermal crystallization temperature on a novel FDU-12 mesoporous composite assembled by ZSM-5 nanoclusters and its hydrodesulfurization performance for DBT and FCC diesel, Fuel Processing Technology 2018, 180, 56–66.
42	NiMo/ZF-140	10.1016/j.fuproc.2018.08.010	Li, Y. Y.; Chi, K. B.; Zhang, H. L.; Du, P.; Hu, D.; Xiao, C. K.; Li, H. P.; Zhao, Z.; Duan, A. J.; Xu, C. M. The influence of hydrothermal crystallization temperature on a novel FDU-12 mesoporous composite assembled by ZSM-5 nanoclusters

			and its hydrodesulfurization performance for DBT and FCC diesel, Fuel Processing Technology 2018, 180, 56–66.
43	NiMo/ZrAT-5	10.1021/acs.iecr.8b01214	Wang, B.; Xiao, C. K.; Li, P. F.; Zhao, Z. S.; Xu, C. M.; Zhao, Z.; Meng, Q.; Li, J. M.; Duan, A. J.; Chen, Z. T. Hydrotreating Performance of FCC Diesel and Dibenzothiophene over NiMo Supported Zirconium Modified Al-TUD-1 Catalysts, Ind. Eng. Chem. Res. 2018, 57, 11868?11882.
44	NiMo/ZrAT-10	10.1021/acs.iecr.8b01214	Wang, B.; Xiao, C. K.; Li, P. F.; Zhao, Z. S.; Xu, C. M.; Zhao, Z.; Meng, Q.; Li, J. M.; Duan, A. J.; Chen, Z. T. Hydrotreating Performance of FCC Diesel and Dibenzothiophene over NiMo Supported Zirconium Modified Al-TUD-1 Catalysts, Ind. Eng. Chem. Res. 2018, 57, 11868?11882.
45	NiMo/ZrAT-25	10.1021/acs.iecr.8b01214	Wang, B.; Xiao, C. K.; Li, P. F.; Zhao, Z. S.; Xu, C. M.; Zhao, Z.; Meng, Q.; Li, J. M.; Duan, A. J.; Chen, Z. T. Hydrotreating Performance of FCC Diesel and Dibenzothiophene over NiMo Supported Zirconium Modified Al-TUD-1 Catalysts, Ind. Eng. Chem. Res. 2018, 57, 11868?11882.
46	NiMo/ZrAT-50	10.1021/acs.iecr.8b01214	Wang, B.; Xiao, C. K.; Li, P. F.; Zhao, Z. S.; Xu, C. M.; Zhao, Z.; Meng, Q.; Li, J. M.; Duan, A. J.; Chen, Z. T. Hydrotreating Performance of FCC Diesel and Dibenzothiophene over NiMo Supported Zirconium Modified Al-TUD-1 Catalysts, Ind. Eng. Chem. Res. 2018, 57, 11868?11882.
47	NiMo/ZrAT-100	10.1021/acs.iecr.8b01214	Wang, B.; Xiao, C. K.; Li, P. F.; Zhao, Z. S.; Xu, C. M.; Zhao, Z.; Meng, Q.; Li, J. M.; Duan, A. J.; Chen, Z. T. Hydrotreating Performance of FCC Diesel and Dibenzothiophene over NiMo Supported Zirconium Modified Al-TUD-1 Catalysts, Ind. Eng. Chem. Res. 2018, 57, 11868?11882.
48	NiMo/AT-100	10.1021/acs.iecr.8b01214	Wang, B.; Xiao, C. K.; Li, P. F.; Zhao, Z. S.; Xu, C. M.; Zhao, Z.; Meng, Q.; Li, J. M.; Duan, A. J.; Chen, Z. T. Hydrotreating Performance of FCC Diesel and Dibenzothiophene over NiMo Supported Zirconium Modified Al-TUD-1 Catalysts, Ind. Eng. Chem. Res. 2018, 57, 11868?11882.
49	NiMo/ZS-1	10.1021/acscatal.7b04147	Wang, X. L.; Mei, J. L.; Zhao, Z.; Zheng, P.; Chen, Z. T.; Gao, D. W.; Fu, J. Y.; Fan, J. Y.; Duan, A. J.; Xu, C. M. Self-Assembly of Hierarchically Porous ZSM-5/SBA-16 with Different Morphologies and Its High Isomerization Performance for Hydrodesulfurization of Dibenzothiophene and 4,6-Dimethyldibenzothiophene, ACS Catal. 2018, 8, 1891?1902.
50	NiMo/ZS-2	10.1021/acscatal.7b04147	Wang, X. L.; Mei, J. L.; Zhao, Z.; Zheng, P.; Chen, Z. T.; Gao, D. W.; Fu, J. Y.; Fan, J. Y.; Duan, A. J.; Xu, C. M. Self-Assembly of Hierarchically Porous ZSM-5/SBA-16 with Different Morphologies and Its High Isomerization Performance for Hydrodesulfurization of Dibenzothiophene and 4,6-Dimethyldibenzothiophene, ACS Catal. 2018, 8, 1891?1902.
51	NiMo/ZS-3	10.1021/acscatal.7b04147	Wang, X. L.; Mei, J. L.; Zhao, Z.; Zheng, P.; Chen, Z. T.; Gao, D. W.; Fu, J. Y.; Fan, J. Y.; Duan, A. J.; Xu, C. M. Self-Assembly of Hierarchically Porous ZSM-5/SBA-16 with Different Morphologies and Its High Isomerization Performance for Hydrodesulfurization of Dibenzothiophene and 4,6-Dimethyldibenzothiophene, ACS Catal. 2018, 8, 1891?1902.
52	NiMo/ZS-4	10.1021/acscatal.7b04147	Wang, X. L.; Mei, J. L.; Zhao, Z.; Zheng, P.; Chen, Z. T.; Gao, D. W.; Fu, J. Y.; Fan, J. Y.; Duan, A. J.; Xu, C. M. Self-Assembly of Hierarchically Porous ZSM-5/SBA-16 with Different Morphologies and Its High Isomerization Performance for Hydrodesulfurization of Dibenzothiophene and 4,6-Dimethyldibenzothiophene, ACS Catal. 2018, 8, 1891?1902.

53	NiMo/ZS-5	10.1021/acscatal.7b04147	Wang, X. L.; Mei, J. L.; Zhao, Z.; Zheng, P.; Chen, Z. T.; Gao, D. W.; Fu, J. Y.; Fan, J. Y.; Duan, A. J.; Xu, C. M. Self-Assembly of Hierarchically Porous ZSM-5/SBA-16 with Different Morphologies and Its High Isomerization Performance for Hydrodesulfurization of Dibenzothiophene and 4,6-Dimethyldibenzothiophene, ACS Catal. 2018, 8, 1891?1902.
54	CoMo submicrotube	10.1039/c7cy01724c	Li, G. C.; Yue, L.; Fan, R. K.; Liu, D.; Li, X. B. Synthesis of a Co-Mo sulfide catalyst with a hollow structure for highly efficient hydrodesulfurization of dibenzothiophene, Catal. Sci. Technol., 2017,7, 5505-5509.
55	NiMo/TiSBA-15	10.1016/j.fuel.2017.01.007	Morales-Ortuño, J. C.; Klimova, T. E., Development of new hydrodesulfurization NiMo catalysts supported on Al2O3-TiSBA-15 hybrid materials, Fuel, 2017, 198, 99-109.
56	NiMo/ATS(60)	10.1016/j.fuel.2017.01.007	Morales-Ortuño, J. C.; Klimova, T. E., Development of new hydrodesulfurization NiMo catalysts supported on Al2O3-TiSBA-15 hybrid materials, Fuel, 2017, 198, 99-109.
57	NiMo/ATS(40)	10.1016/j.fuel.2017.01.007	Morales-Ortuño, J. C.; Klimova, T. E., Development of new hydrodesulfurization NiMo catalysts supported on Al2O3-TiSBA-15 hybrid materials, Fuel, 2017, 198, 99-109.
58	NiMo/ATS(20)	10.1016/j.fuel.2017.01.007	Morales-Ortuño, J. C.; Klimova, T. E., Development of new hydrodesulfurization NiMo catalysts supported on Al2O3-TiSBA-15 hybrid materials, Fuel, 2017, 198, 99-109.
59	NiMo/gamma-Al2O3	10.1016/j.fuel.2017.01.007	Morales-Ortuño, J. C.; Klimova, T. E., Development of new hydrodesulfurization NiMo catalysts supported on Al2O3-TiSBA-15 hybrid materials, Fuel, 2017, 198, 99-109.
60	NiMo/Ti-SBA-15	10.1016/j.cattod.2015.07.028	Morales-Ortuño, J. C.; Ortega-Dominguez, R. A.; Hernandez-Hipolito, P.; Bokhimi, X.; Klimova, T. E., HDS performance of NiMo catalysts supported on nanostructured materials containing titania, Catalysis Today 2016, 271, 127–139.
61	NiMo/Ti-SBA-16	10.1016/j.cattod.2015.07.028	Morales-Ortuño, J. C.; Ortega-Dominguez, R. A.; Hernandez-Hipolito, P.; Bokhimi, X.; Klimova, T. E., HDS performance of NiMo catalysts supported on nanostructured materials containing titania, Catalysis Today 2016, 271, 127–139.
62	NiMo/NT(3)	10.1016/j.cattod.2015.07.028	Morales-Ortuño, J. C.; Ortega-Dominguez, R. A.; Hernandez-Hipolito, P.; Bokhimi, X.; Klimova, T. E., HDS performance of NiMo catalysts supported on nanostructured materials containing titania, Catalysis Today 2016, 271, 127–139.
63	NiMo/NT(5)	10.1016/j.cattod.2015.07.028	Morales-Ortuño, J. C.; Ortega-Dominguez, R. A.; Hernandez-Hipolito, P.; Bokhimi, X.; Klimova, T. E., HDS performance of NiMo catalysts supported on nanostructured materials containing titania, Catalysis Today 2016, 271, 127–139.
64	NiMo/gamma-Al2P3	10.1016/j.cattod.2015.07.028	Morales-Ortuño, J. C.; Ortega-Dominguez, R. A.; Hernandez-Hipolito, P.; Bokhimi, X.; Klimova, T. E., HDS performance of NiMo catalysts supported on nanostructured materials containing titania, Catalysis Today 2016, 271, 127–139.
65	DTAB/Mo(0)	10.1016/j.apcata.2016.06.017	Yuan, P.; Cui, C. S.; Han, W.; Bao, X. J. The preparation of Mo/gamma-Al2O3 catalysts with controllable size and morphology via adjusting the metal-support interaction and their hydrodesulfurization performance, Applied Catalysis A:

			General 2016, 524, 115–125.
66	DTAB/Mo(1/7)	10.1016/j.apcata.2016.06.017	Yuan, P.; Cui, C. S.; Han, W.; Bao, X. J. The preparation of Mo/gamma-Al2O3 catalysts with controllable size and morphology via adjusting the metal-support interaction and their hydrodesulfurization performance, Applied Catalysis A: General 2016, 524, 115–125.
67	DTAB/Mo(3/5)	10.1016/j.apcata.2016.06.017	Yuan, P.; Cui, C. S.; Han, W.; Bao, X. J. The preparation of Mo/gamma-Al2O3 catalysts with controllable size and morphology via adjusting the metal-support interaction and their hydrodesulfurization performance, Applied Catalysis A: General 2016, 524, 115–125.
68	DTAB/Mo(6/7)	10.1016/j.apcata.2016.06.017	Yuan, P.; Cui, C. S.; Han, W.; Bao, X. J. The preparation of Mo/gamma-Al2O3 catalysts with controllable size and morphology via adjusting the metal-support interaction and their hydrodesulfurization performance, Applied Catalysis A: General 2016, 524, 115–125.
69	DTAB/Mo(7/6)	10.1016/j.apcata.2016.06.017	Yuan, P.; Cui, C. S.; Han, W.; Bao, X. J. The preparation of Mo/gamma-Al2O3 catalysts with controllable size and morphology via adjusting the metal-support interaction and their hydrodesulfurization performance, Applied Catalysis A: General 2016, 524, 115–125.
70	NiMoW/Al2O3	10.1016/j.cattod.2014.05.002	Mendoza-Nieto, J. A.; Robles-Mendez, F.; Klimova, T. E. Support effect on the catalytic performance of trimetallic NiMoW catalysts prepared with citric acid in HDS of dibenzothiophenes, Catalysis Today, 2015, 250, 47?59.
71	NiMoW(1)/Al2O3	10.1016/j.cattod.2014.05.002	Mendoza-Nieto, J. A.; Robles-Mendez, F.; Klimova, T. E. Support effect on the catalytic performance of trimetallic NiMoW catalysts prepared with citric acid in HDS of dibenzothiophenes, Catalysis Today, 2015, 250, 47?59.
72	NiMoW(9)/Al2O3	10.1016/j.cattod.2014.05.002	Mendoza-Nieto, J. A.; Robles-Mendez, F.; Klimova, T. E. Support effect on the catalytic performance of trimetallic NiMoW catalysts prepared with citric acid in HDS of dibenzothiophenes, Catalysis Today, 2015, 250, 47?59.
73	NiMoW/SBA-15	10.1016/j.cattod.2014.05.002	Mendoza-Nieto, J. A.; Robles-Mendez, F.; Klimova, T. E. Support effect on the catalytic performance of trimetallic NiMoW catalysts prepared with citric acid in HDS of dibenzothiophenes, Catalysis Today, 2015, 250, 47?59.
74	NiMoW(1)/SBA-15	10.1016/j.cattod.2014.05.002	Mendoza-Nieto, J. A.; Robles-Mendez, F.; Klimova, T. E. Support effect on the catalytic performance of trimetallic NiMoW catalysts prepared with citric acid in HDS of dibenzothiophenes, Catalysis Today, 2015, 250, 47?59.
75	NiMoW(9)/SBA-15	10.1016/j.cattod.2014.05.002	Mendoza-Nieto, J. A.; Robles-Mendez, F.; Klimova, T. E. Support effect on the catalytic performance of trimetallic NiMoW catalysts prepared with citric acid in HDS of dibenzothiophenes, Catalysis Today, 2015, 250, 47?59.
76	NiMo	10.1016/j.cattod.2013.06.002	Calderon-Magdaleno, M. A.; Mendoza-Nieto, J. A.; Klimova, T. E. Effect of the amount of citric acid used in the preparation of NiMo/SBA-15 catalysts on their performance in HDS of dibenzothiophene-type compounds, Catalysis Today 2014, 220–222, 78–88.
77	NiMoCA(0.5)	10.1016/j.cattod.2013.06.002	Calderon-Magdaleno, M. A.; Mendoza-Nieto, J. A.; Klimova, T. E. Effect of the amount of citric acid used in the preparation of NiMo/SBA-15 catalysts on their performance in HDS of dibenzothiophene-type compounds, Catalysis Today 2014, 220–222, 78–88.
78	NiMoCA(1.0)	10.1016/j.cattod.2013.06.002	Calderon-Magdaleno, M. A.; Mendoza-Nieto, J. A.; Klimova, T. E. Effect of the amount of citric acid used in the preparation of NiMo/SBA-15 catalysts on their performance in HDS of dibenzothiophene-type compounds, Catalysis Today

			2014, 220–222, 78–88.
79	NiMoCA(1.5)	10.1016/j.cattod.2013.06.002	Calderon-Magdaleno, M. A.; Mendoza-Nieto, J. A.; Klimova, T. E. Effect of the amount of citric acid used in the preparation of NiMo/SBA-15 catalysts on their performance in HDS of dibenzothiophene-type compounds, Catalysis Today 2014, 220–222, 78–88.
80	NiMoCA(2.0)	10.1016/j.cattod.2013.06.002	Calderon-Magdaleno, M. A.; Mendoza-Nieto, J. A.; Klimova, T. E. Effect of the amount of citric acid used in the preparation of NiMo/SBA-15 catalysts on their performance in HDS of dibenzothiophene-type compounds, Catalysis Today 2014, 220–222, 78–88.
81	NiMo(d)	10.1016/j.jcat.2013.03.027	Klimova, T. E.; Valencia, D.; Mendoza-Nieto, J. A.; Hernandez-Hipolito, P. Behavior of NiMo/SBA-15 catalysts prepared with citric acid in simultaneous hydrodesulfurization of dibenzothiophene and 4,6-dimethyldibenzothiophene, Journal of Catalysis, 2013, 304, 29-46.
82	NiMo(c)	10.1016/j.jcat.2013.03.027	Klimova, T. E.; Valencia, D.; Mendoza-Nieto, J. A.; Hernandez-Hipolito, P. Behavior of NiMo/SBA-15 catalysts prepared with citric acid in simultaneous hydrodesulfurization of dibenzothiophene and 4,6-dimethyldibenzothiophene, Journal of Catalysis, 2013, 304, 29-46.
83	NiMoCA(1-d)	10.1016/j.jcat.2013.03.027	Klimova, T. E.; Valencia, D.; Mendoza-Nieto, J. A.; Hernandez-Hipolito, P. Behavior of NiMo/SBA-15 catalysts prepared with citric acid in simultaneous hydrodesulfurization of dibenzothiophene and 4,6-dimethyldibenzothiophene, Journal of Catalysis, 2013, 304, 29-46.
84	NiMoCA(1-c)	10.1016/j.jcat.2013.03.027	Klimova, T. E.; Valencia, D.; Mendoza-Nieto, J. A.; Hernandez-Hipolito, P. Behavior of NiMo/SBA-15 catalysts prepared with citric acid in simultaneous hydrodesulfurization of dibenzothiophene and 4,6-dimethyldibenzothiophene, Journal of Catalysis, 2013, 304, 29-46.
85	NiMoCA(9-d)	10.1016/j.jcat.2013.03.027	Klimova, T. E.; Valencia, D.; Mendoza-Nieto, J. A.; Hernandez-Hipolito, P. Behavior of NiMo/SBA-15 catalysts prepared with citric acid in simultaneous hydrodesulfurization of dibenzothiophene and 4,6-dimethyldibenzothiophene, Journal of Catalysis, 2013, 304, 29-46.
86	NiMoCA(9-c)	10.1016/j.jcat.2013.03.027	Klimova, T. E.; Valencia, D.; Mendoza-Nieto, J. A.; Hernandez-Hipolito, P. Behavior of NiMo/SBA-15 catalysts prepared with citric acid in simultaneous hydrodesulfurization of dibenzothiophene and 4,6-dimethyldibenzothiophene, Journal of Catalysis, 2013, 304, 29-46.

Figure S1. Frequency plots of catalyst descriptors.

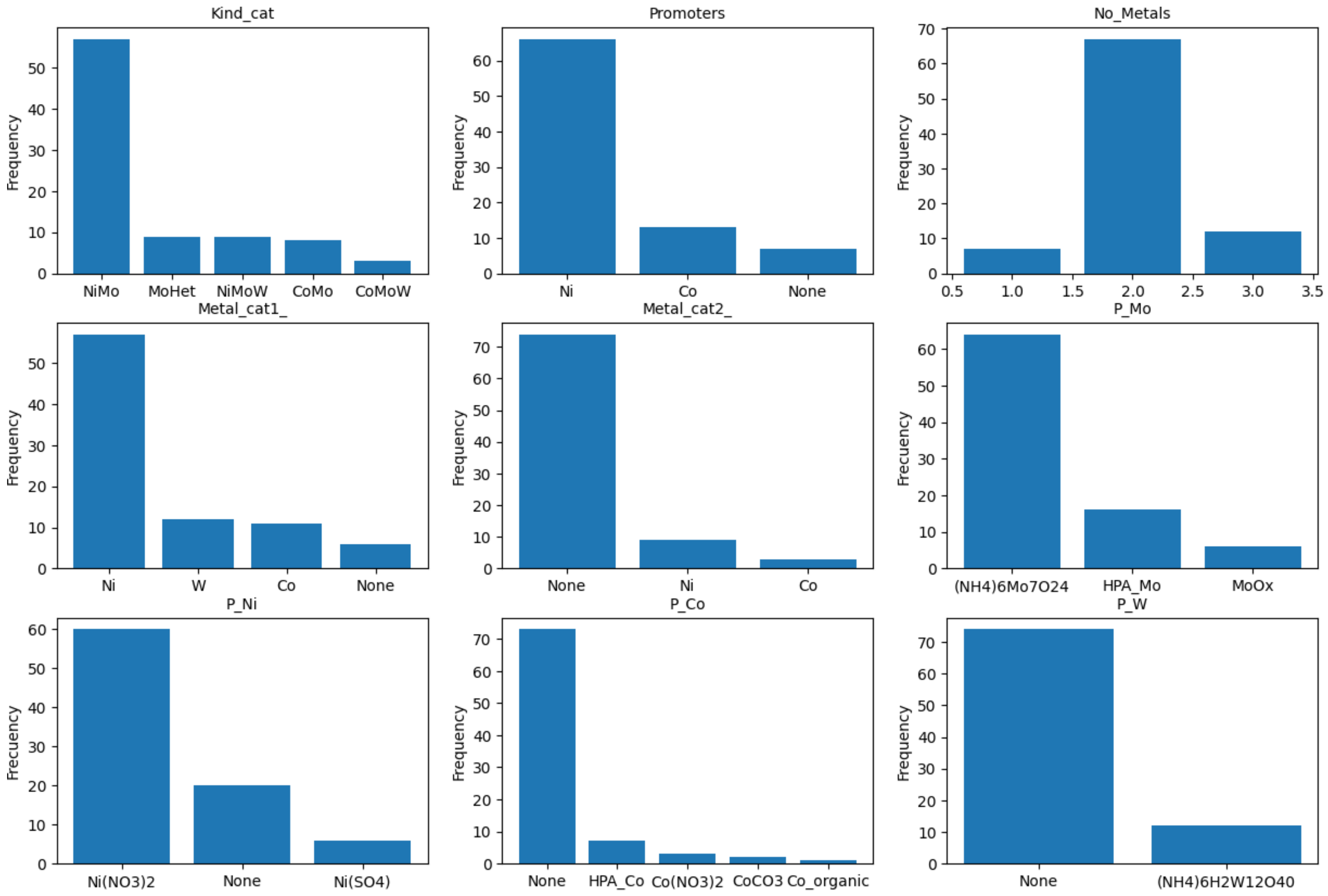

Figure S2. Frequency plots of support descriptors.

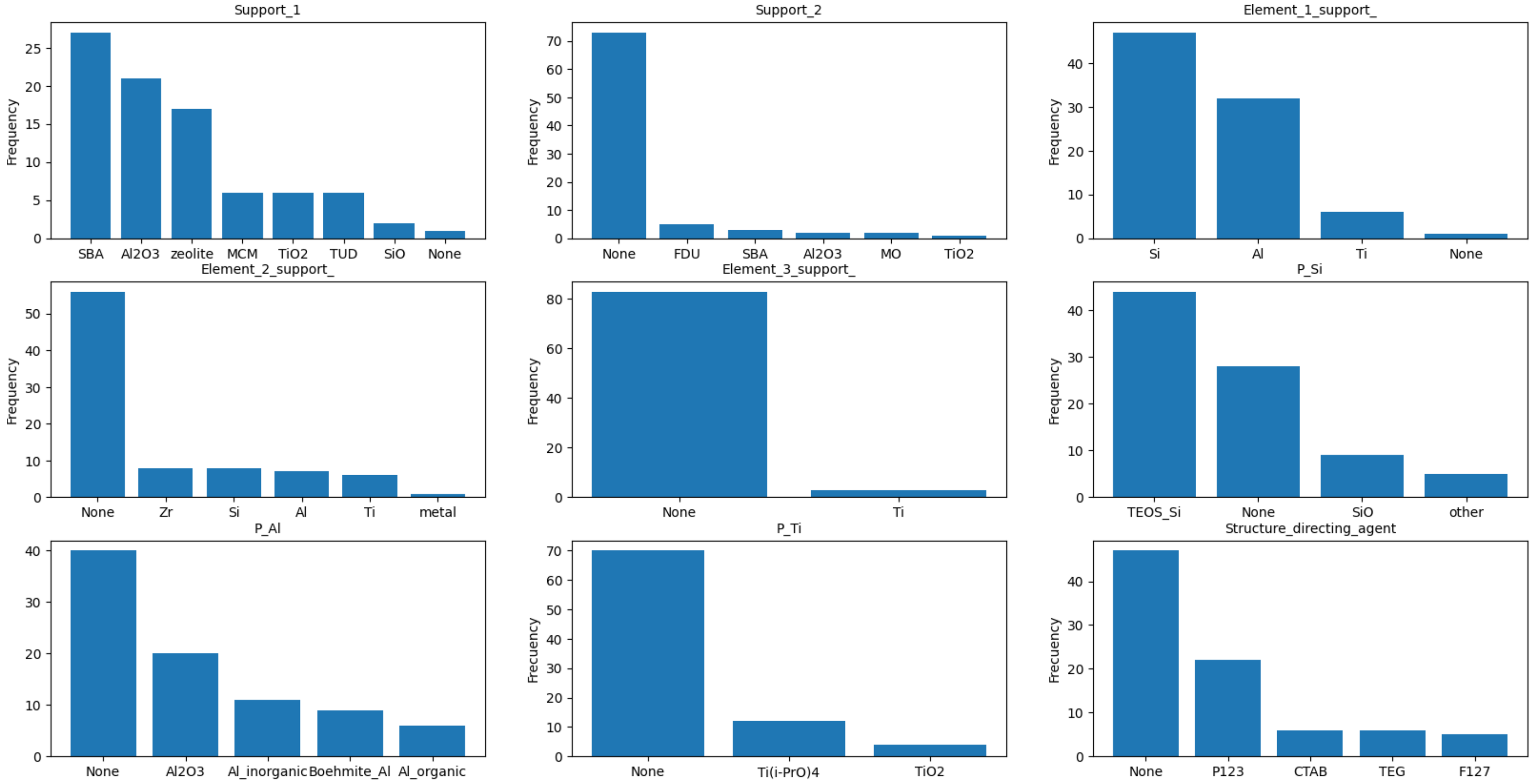

Figure S3. Frequency plots of other descriptors.

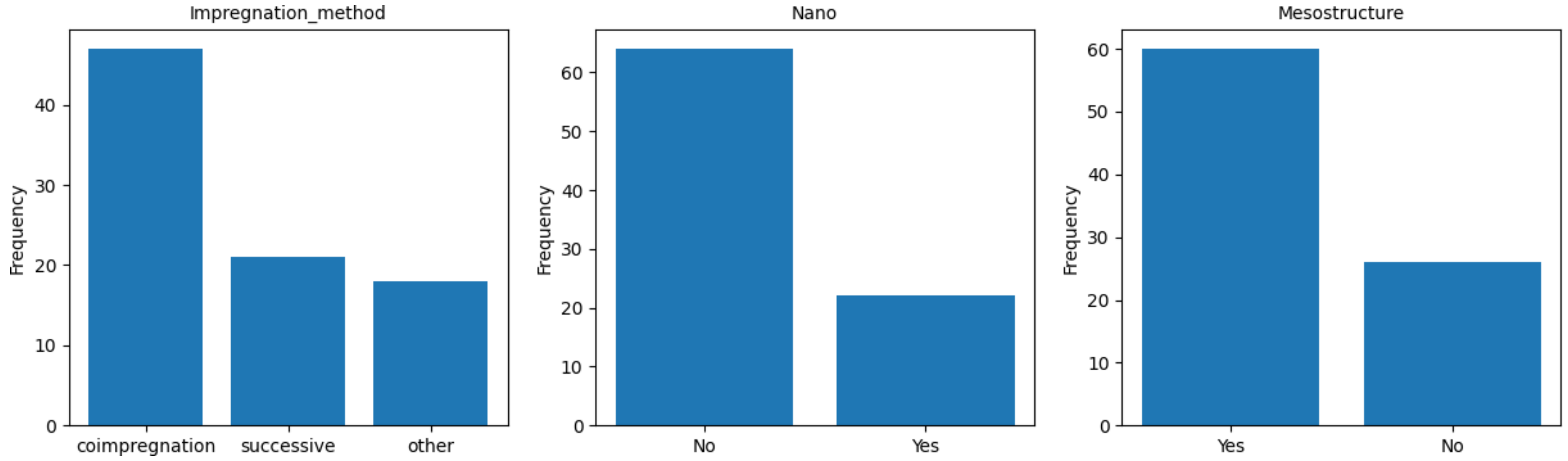

Figure S4. Histogram of numerical descriptors.

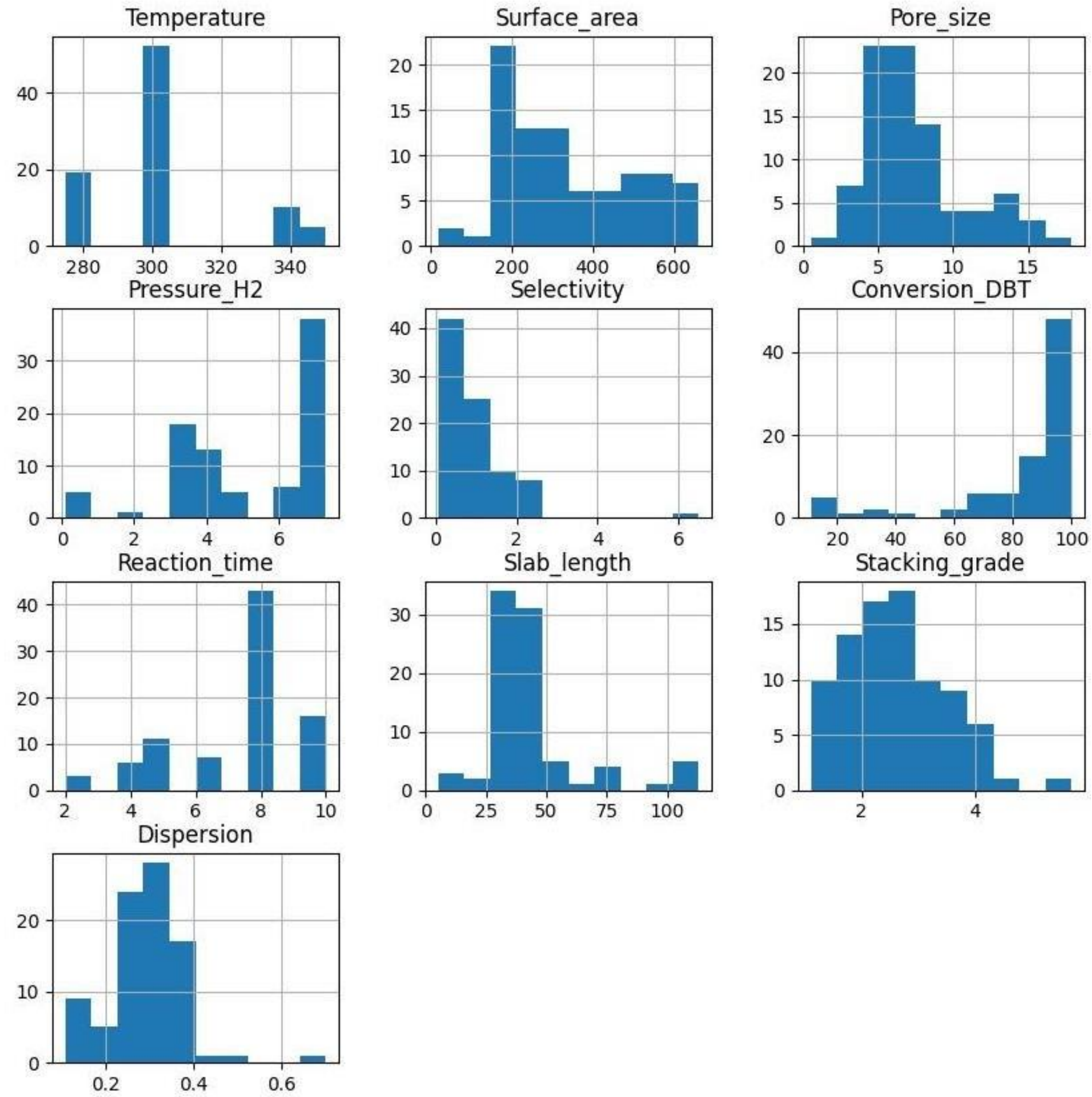

Table S2. Coefficients of Lasso and Ridge regressions for estimation of conversion of DBT.

Predictors	Ridge Coefficients	Lasso Coefficients
Kind_cat	0.5229	0.6098
Temperature	0.3878	0.3850
P_Si	0.2260	0.2358
P_Al	0.1418	0.1518
Stacking_grade	0.1430	0.1468
Nano	0.1319	0.1324
Structure_directing_agent	0.1376	0.1254
P_W	0.0611	0.0867
Impregnation_method	0.0644	0.0425
Mesostructure	0.0432	0.0234
Element_1_support_	0.0648	0.0112
Pressure_H2	0.0371	0.0043
No_Metals	0.0163	0.0000
Metal_cat2_	0.0589	0.0000
Element_3_support_	0.0367	0.0000
Selectivity	0.0065	0.0000
Slab_length	-0.0377	0.0000
Promoters	0.0336	0.0000
P_Mo	0.0085	0.0000
Dispersion	-0.0539	-0.0002
Surface_area	-0.0809	-0.0316
Element_2_support_	-0.0753	-0.0402
Support_2	-0.0378	-0.0668
Pore_size	-0.1549	-0.1057
P_Ti	-0.0936	-0.1147
Support_1	-0.1847	-0.1273
P_Ni	-0.1828	-0.1331

Metal_cat1_	-0.1891	-0.1887
Aditive	-0.2262	-0.2247
Reaction_time	-0.5110	-0.5210
P_Co	-0.5501	-0.5298
β_0	1.1505	1.1376

Table S3. Coefficients of Lasso and Ridge regressions for estimation of selectivity.

predictor	Ridge Coefficients	Lasso Coefficients
Element_1_support_	0.3129	0.4003
Kind_cat	0.2297	0.3031
Promoters	0.2289	0.2880
Slab_length	0.1700	0.1545
P_Co	0.1935	0.1025
Surface_area	0.1423	0.0911
Impregnation_method	0.0782	0.0901
Stacking_grade	0.0684	0.0879
Metal_cat1_	0.0727	0.0496
Mesostructure	0.0656	0.0385
Support_1	0.0261	0.0113
Nano	0.0492	0.0113
Element_3_support_	0.0627	0.0050
No_Metals	0.0127	0.0000
Element_2_support_	0.0402	0.0000
Pressure_H2	-0.0302	0.0000
Conversion_DBT	-0.0319	0.0000
Reaction_time	-0.0643	0.0000
Aditive	-0.0036	0.0000
Support_2	-0.0448	0.0000
P_Ni	0.0174	0.0000
P_W	-0.0190	0.0000
P_Si	0.0280	0.0000
P_Mo	-0.0692	-0.0200
P_Ti	-0.0929	-0.0327
Metal_cat2_	-0.0642	-0.0546
Dispersion	-0.0954	-0.0639

P_Al	-0.1117	-0.1411
Structure_directing_agent	-0.1580	-0.1639
Temperature	-0.3731	-0.4242
Pore_size	-0.3976	-0.4450
β_0	0.2267	0.2217
